# Efficacy of early oral refeeding in patients of mild acute pancreatitis

**DOI:** 10.12669/pjms.334.12338

**Published:** 2017

**Authors:** Shahum Khan, Waqas Ahmed Ranjha, Hassan Tariq, Hareem Nawaz

**Affiliations:** 1 Dr. Shahum Khan, MBBS, Department of Surgery, CMH Rawalpindi, Pakistan; 2 Dr. Waqas Ahmed Ranjha, MBBS, Department of Surgery, CMH Rawalpindi, Pakistan; 3 Dr. Hassan Tariq, MBBS, Armed Forces Institute of Pathology, Rawalpindi, Pakistan; 4 Dr. Hareem Nawaz, MBBS, Department of Radiology, CMH Kharian, Pakistan

**Keywords:** Acute Pancreatitis, Early and Late Oral Refeeding, Length Of hospital stay

## Abstract

**Objective:**

To compare Early Oral Refeeding (EORF) with Routine Oral Refeeding (RORF) on outcome of patients of mild Acute Pancreatitis (AP) in terms of Mean Length of Hospital Stay (LOHS).

**Methods:**

This randomized controlled trialwas conducted atSurgical Department CMH Rawalpindi, from 1st Feb 2015 to 01st Aug 2016. A total of 60 patients with pain epigastrium were enrolled in the study. Severity of pancreatitis was assessed using Glasgow Scale. Patients were randomly divided in two groups. Group-A was started feeding within 12 hours (EORF group) and Group-B after 12 hours (RORF group). Demographic details and data were recorded on a structured proforma. After discharge, LOHS was measured for both groups and outcome was compared.

**Results:**

The groups were comparable with respect to age, sex, etiology, Glasgow Scale, time from onset of pain and Serum Amylase levels at admission. Treatment was standardized according to international guidelines for both groups. The mean LOHS was 7.8 ± 2.14 days in the Group-A and 10.03 ± 1.75 days in Group-B. The difference in the mean LOHS between the two groups was statistically significant (p<0.05).

**Conclusion:**

In patients of mild acute pancreatitis, early oral feeding is feasible and safe and has better outcome then those with routine oral refeeding.

## INTRODUCTION

Acute Pancreatitis (AP) is an inflammatory process of pancreas that presents with different severity degrees, ranging from a mild self-limited disease, with interstitial edema in the pancreas, to a severe disease with extensive necrosis.^1^ Pancreatic rest by Nil Per Oral(NPO) strategy is considered necessary in AP till abdominal pain get resolved and the levels of pancreatic and inflammatory markers decrease.^2^

Overall, in about 15% to 20% of patients, AP progresses to a severe illness with a prolonged disease course.^3^ These severely ill patients may develop organ failure and / or local complications such as pancreatic necrosis.^4^ Approximately 75% of the patients have mild disease with mortality below 1%.^5^ Mortality increases up to 20% if the disease progresses to its severe necrotizing form,^6^ and in the most severe cases mortality can range from 30 to 40%.^7^ In severe necrotizing pancreatitis, 80% of all patients are catabolic, with high energy expenditure and enhanced protein catabolism. The negative nitrogen balance can be as much as 40 g/day and can have a deleterious effect on both nutritional status and disease progression.^8^-^10^

It is well demonstrated that the damage of gut barrier is responsible for the initiation ofSystemic Inflammatory Response Syndrome (SIRS) and sepsis and associated with the pancreatic necrosis. Gut barrier is damaged in the early phase of AP and intestinal permeability is significantly increased in severe attacks of AP within 72 hours.^11^ Pancreatic rest through NPO was always considered necessary in AP and the general practice is to start oral refeeding in three to seven days of hospitalization. Enteral Nutrition (EN) in recent researcheshas found the ability to maintain gut integrity, stimulate gut contractility and the release of immunomodulation agents and blood flow to the gut. Maintaining mucosal integrity reduces the release of inflammatory mediators, decreases oxidative stress and abates the SIRS.^12^

Though it is clear that long-lasting Total Parenteral Nutrition (TPN) or total “gut rest” brings no benefits, questions about whether there is no need for “gut rest” at all and whether oral re-feeding is better in the very early phase of AP are being asked.^13^ This paucity of data is a serious concern for lot of physicians. Unfortunately, past evidences are rare concerning when to start optimal refeeding after onset of mild AP.^14^

In a recent research, a total of 149 patients were evaluated for time interval between disease onset and initiation of oral refeeding, Total Length of Hospital Stay (LOHS), post refeeding LOHS, and adverse Gastro-Intestinal (GI) events.^15^ Patients in the Early Oral Refeeding (EORF) group started refeeding significantly earlier than those in the Routine Oral Refeeding (RORF) group. Moreover, patients in the EORF grouphad significantly shorter total LOHS (6.8 ± 2.1 vs. 10.4 ± 4.1 days; P < 0.01). There was no significant difference in adverse gastrointestinal events between the two groups. Theaims of study was to compare outcome of EORF on early recovery of patients of mildAP withRORF protocol in hospitals through LOHS.

## METHODS

This randomized controlled trial was carried out in department of surgery CMH Rawalpindi, Pakistan from Feb 2015 to Aug 2015. A Total number of 60 patients were included in study. Sample size was measured using WHO calculator with confidence interval of 95% along with significance level of 5%, keeping power of test at 90%. After approval from hospital ethical committee, patients between 20 to 70 years of age, presenting in the emergency department and outpatient department with GI)symptoms of pain epigastrium, nausea and vomiting and Serum Amylase level of more than 200 IU/L (Twice of normal value of 100 IU/L), thus meeting admission criteria of AP were admitted in hospital. Patients with mild AP considered in terms of Glasgow prognostic score of less thanthree, and pain assessment of less than four On Visual Analogue Scale (VAS), post- Endoscopic Retrograde Cholangiopancreatography (ERCP) AP, history of Cholelithiasis and not requiring emergency admission, post blunt trauma pancreatitis and patients of alcoholic pancreatitis were included. Patients with post-penetrating trauma pancreatitis, post burn pancreatitis, obstructive jaundice, and pancreatitis with choledocholithiasis, acid peptic disease with perforated duodenum, carcinoma pancreas, esophageal perforation, mumps and pregnancy were excluded. Patients were divided into Group-A and Group-B using consecutive non-probability sampling technique. Each group comprised of 30 Patients. Group-A consisted of patients on EORF i.e. feeding started within 12hours of presentation and Group-B consisted of those on RORF mainly after 12 hours of presentation. Informed written consent was taken from every patient regarding participation. Demographic details and above mentioned details were recorded on a structured proforma. Patient with subsequently normal Serum Amylase (less than 100 IU/l) and relieved GI symptoms were discharged. After discharge, LOHS was measured for both groups.

### Statistical Analysis

All the data was entered and analyzed using SPSS Version 20. Quantitative variables like age and LOHS were measured as mean and standard deviation. Effect modifiers like age and gender were controlled by stratification. Post stratification, independent sample t-test were used to compare LOHS between two groups. P value of ≤ 0.05 was considered statistically significant.

## RESULTS

A total of 60 patients were included in this study. Out of these, 36 (60%) were males, and24 (40%) were females. Mean age of study population was 44.10±12.72 years (Range 23-68 years). Out of the 60 patients, 29 (48.3%) patients had mild AP, 25 (41.7%) had gallstone pancreatitis, 3 (5%) patients had post blunt trauma pancreatitis and 3 (5%) had Post ERCP AP. Gender wise distribution of etiology of AP is illustrated in Table-I. Mild AP was more seen in male patients and more gallstone pancreatitis was seen in females. The group-wise statistics indicating LOHS is given in Table-II. The difference in LOHS between two groups was statistically significant, with the patients receiving EORF having less hospital stay as compared to those of RORF.

**Table-I:** Type of illness amongst patients with respect to gender (n=60).

*Variable*		*Type of Illness*				*Total*

		*Acute Mild Pancreatitis (n)*	*Gallstone Pancreatitis (n)*	*Post Traumatic Pancreatitis (n)*	*Post ERCP Acute Mild Pancreatitis (n)*
Gender	Male	22	10	2	2	36
	Female	7	15	1	1	24

Total		29	25	3	3	60

**Table-II:** Comparison of LOHS between two Groups (n=60).

*Oral Refeeding of Patients*	*n*	*LOHS (Days) Mean ± SD*	*p Value*
RORF	30	10.03±1.752	0.001 (p<0.05)
EORF	30	7.80±2.140	

## DISCUSSION

Early enteral feeding is accepted in the treatment of AP,^16^ at the same time, EORF is deemed to be detrimental in the early phase of AP. For decades, NPO was the common strategy in the treatment of AP. The main argument against oral feeding was fear of possible augmentation of the autodigestive process in the pancreatic gland and peripancreatic tissue. This could happen due to the stimulatory effect from the oral food intake to the pancreatic exocrine secretion^4^. The usual criteria for re-initiating oral feeding are the absence of abdominal pain, nausea and vomiting, restoration of appetite and normalization of laboratory findings including serum amylase and lipase levels. Data to support EORF without normalization of serum parameters are increasingly convincing. The results of an open randomized multicenter trial suggested that normalization of serum lipase levels is not obligatory before reinitiating EN in patients with mild AP.^17^

The international consensus guidelines on nutrition therapy for AP make a few key proposals.^18^ First, nutrition support therapy is generally not required for patients with mild to moderate AP, and can be reserved for patients with severe AP. Second, EN is preferred to Parenteral Nutrition (PN) used only when EN is contraindicated or not feasible. Despite such guidelines, lot of discrepancies and confusion still exist between clinical practice and results actually obtained from studies on pancreatic rest, efficacy and safety of early oral refeeding in AP due to scarcity of data.^19^-^22^

In present study, we investigated a total of 60 patients of Mild acute pancreatitis for effect of early oral refeeding and then assess its efficacy and safety in disease management by calculating Total length of hospital stay of patients. Early feeding was based on timings i.e. 12 hours from initial presentation and subjective hunger, without the remission of abdominal pain or normalization of pancreatic amylase and lipase. The group statistics indicated that the mean LOHS for patients who received EORF was 7.8 ± 2.14 days, as compared to 10.03 ± 1.75 days for patients who received RORF. These results are comparable to study by Xian L et al who carried out randomized control trial on 146 patients and found out that total LOHS was significantly shorter in the EORF group than in the RORF group (13.7 ± 5.4 days versus 15.7 ± 6.2 days; P = 0.0398).^23^ A major concern of EORF in AP is premature oral refeeding in patients with different etiologies with intolerance to the reintroduced diet, which can cause AP relapse and prolonged hospital LOS. A meta-analysis by Levy P and co-workers, and other studies reported that intolerance to refeeding occurred in 21% to 25% of patients with AP of different etiologies.^19^-^21^ Our study showed that EORF started within 12 hours of presentation is feasible and safe in different etiologies of patients. Mild AP and same EORF regimen was followed along with same standardization of treatment protocol as per international guidelines.^20^ In a study by Eckerwall GE and colleagues, 60 patients were randomized to the two treatment groups, fasting or immediate oral feeding. No significant difference was seen between the groups concerning levels of amylase, leukocytes, abdominal pain or number of gastrointestinal symptoms. The LOHS was significantly shorter in the oral feeding group, which further aides our study results.^22^

Finally keeping our results in view and their comparison with past studies, we are of the view that EORF is safe and effective in reducing LOHS in patients of mild AP. This treatment modality can be safely implemented in hospitals for betterment of patients.

### Limitations of the Study

The study had few limitations. The VAS was subjective piece of information from the history and physical examination. This study was conducted in single center, so variety of patients couldn’t be assessed. Study population was not a true representation of the society as most of the patients belonged to a particular age group and military background. Estimated daily energy intake and type of intake were not studied. Accurate time at which refeeding should be initiated was not found.

## CONCLUSION

Early oral re-feeding is safe and effective to reduce length of hospital stay in patients of mild acute pancreatitis without aggravating and causing clinical complications in comparison to nil per mouth and routine oral refeeding protocol being followed. Further studies in multiple centers are necessary to confirm the reliability and generalizability of our findings.

### Authors’ Contribution

**SK** conceived and designed the study.

**WAR** did data collection, statistical analysis.

**HT** did statistical analysis and manuscript editing.

**HN** prepared the manuscript.
